# Wall shear stress vectors derived from 3D PC-MRI at increasing resolutions in an intracranial aneurysm phantom

**DOI:** 10.1186/1532-429X-14-S1-W43

**Published:** 2012-02-01

**Authors:** Pim van Ooij, Wouter V Potters, Charles B Majoie, Ed vanBavel, Aart Nederveen

**Affiliations:** 1 Radiology, Academic Medical Center, Amsterdam, Netherlands; 2 Biomedical Engineering & Physics, Academic Medical Center, Amsterdam, Netherlands

## Background

Wall shear stress (WSS) is the friction force that blood flow exerts on the vessel wall. It is thought to affect the function of endothelial cells and the development of atherosclerosis and aneurysms. A promising technique to measure blood flow is three-dimensional phase contrast MRI (3D PC-MRI). Due to limited spatial resolution and SNR, estimating WSS from time-resolved 3D PC-MRI is challenging. In this study, a recently in-house developed WSS algorithm is tested on 3D PC-MRI in an intracranial aneurysm phantom, measured with steady flow at different resolutions.

## Methods

**Figure 1:**
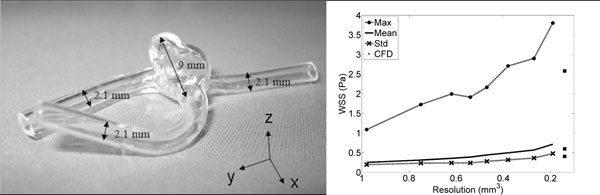
(a) Aneurysm phantom (b) Maximum, mean and standard deviation of the wall shear stress at all resolutions and for CFD

A glass reproduction of a 3DRA of an aneurysm of a patient was manually created and connected to a pump. The phantom with size of 6x4x9 mm^3^ (x, y, z respectively) is shown in figure 1a. A steady (constant flow, water, no gating) PC-MRI measurement was performed on a 3T MR system (Philips Medical System, Best, The Netherlands) in a solenoid rat coil with a diameter of 7 cm at isotropic resolutions starting at 0.2 mm^3^ to 1 mm^3^ with steps of 0.1 mm^3^. TE/TR = 4.28/8.66 ms (0.2 mm^3^ resolution), flip angle: 15°, velocity encoding: 30x60x30 cm/s in the x, y and z direction (see figure 1a) respectively. The CFD geometry was obtained with 3DRA and consisted of 742.316 tetrahedral cells with an average node spacing of 0.14 mm. CFD was performed in FLUENT. The x, y and z components of the inflow as measured with MRI were applied as the inflow boundary conditions. WSS can be calculated by: τ=2ηε.n with τ the WSS vector, η the dynamic viscosity, ε the rate of deformation tensor and n the normal vector in a coordinate at the wall respectively. At each position at the wall a local coordinate system was defined with its z-axis coinciding with the normal vector: n=(0,0,1). By assuming that no flow occurs through the vessel wall, it holds that n.v =0 in the rotated frame. The rate of deformation tensor is then reduced to: ε =(∂v_x_/∂z,∂v_y_/∂z,0). The shear rates ∂v_x_/∂z and ∂v_y_/∂z are obtained from the gradients of the 1D smoothing splines fitted through the x-velocity values and y-velocity values along the direction of the normal.

## Results

**Figure 2:**
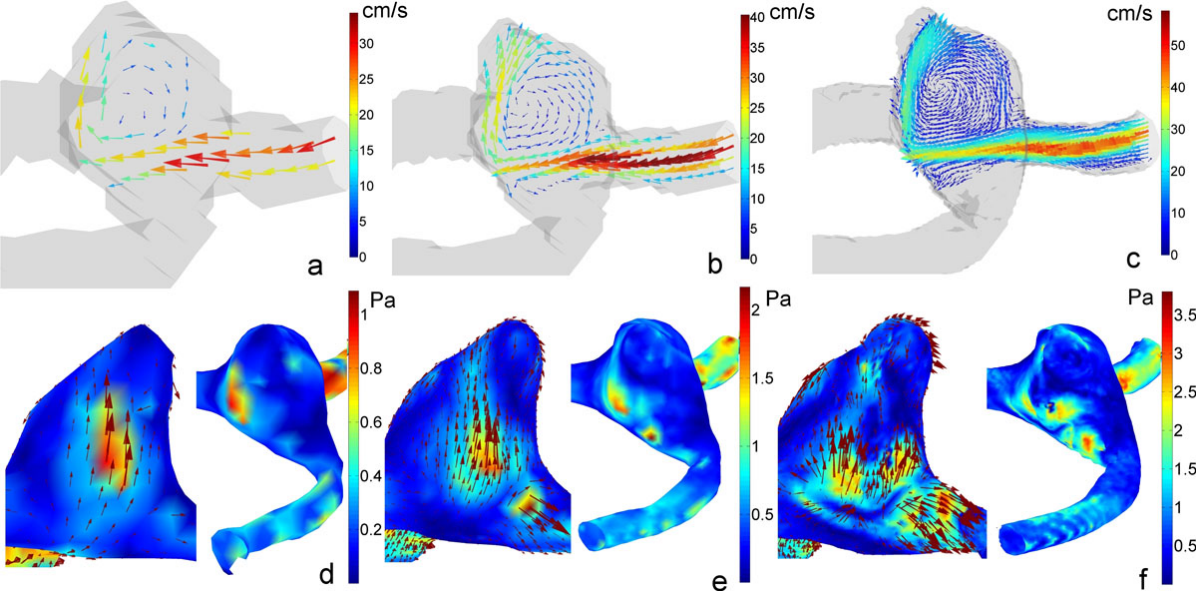
(a) Velocity in a characteristic slice at 1 mm^3^ (b) Velocity in a characteristic slice at 0.5 mm^3^ (c) Velocity in a characteristic slice at 0.2 mm^3^ (d) Wall shear stress at 1 mm^3^ (e) Wall shear stress at 0.5 mm^3^ (f) Wall shear stress at 0.2 mm^3^

The maximum, mean and standard deviation of the WSS increases with increasing resolution (figure 1b). In figure 2a, b and c the direction and magnitude of the velocity vectors in a similar slice measured at 1, 0.5 and 0.2 mm^3^, respectively, are similar. Directions of WSS vectors and regions of high and low WSS in figure 2d, e and f are similar. More WSS details and complexity can be observed in figure 2f than in figures 2d and e. Note in figure 2 that a more accurate segmentation of the phantom is obtained at lower voxel sizes.

## Conclusions

For accurate WSS estimations, resolution and SNR must be as high as possible. This is difficult to attain in clinical 4D PC-MRI protocols. To obtain a qualitative indication of WSS distributions, acquisitions at low resolution may be sufficient.

